# Identifying the Biogeographic Patterns of Rare and Abundant Bacterial Communities Using Different Primer Sets on the Loess Plateau

**DOI:** 10.3390/microorganisms9010139

**Published:** 2021-01-09

**Authors:** Quanchao Zeng, Shaoshan An

**Affiliations:** 1 College of Resources and Environment, Huazhong Agricultural University, Wuhan 430070, China; zengchao256@126.com; 2 State Key Laboratory of Soil Erosion and Dryland Farming on the Loess Plateau, Institute of Soil and Water Conservation, Northwest A&F University, Yangling 712100, China

**Keywords:** primer sets, rare bacteria, spatial patterns, arid regions, land uses

## Abstract

High-throughput sequencing is commonly used to study soil microbial communities. However, different primers targeting different 16S rRNA hypervariable regions often generate different microbial communities and result in different values of diversity and community structure. This study determined the consequences of using two bacterial primers (338f/806r, targeting the V3–V4 region, and 520f/802r, targeting the V4 region) to assess bacterial communities in the soils of different land uses along a latitudinal gradient. The results showed that the variations in the soil bacterial diversity in different land uses were more evident based on the former pair. The statistical results showed that land use had no significant impact on soil bacterial diversity when primer pair 520f/802r was used. In contrast, when primer pair 338f/806r was used, the cropland and orchard soils had significantly higher operational taxonomic units (OTUs) and Shannon diversity index values than those of the shrubland and grassland soils. Similarly, the soil bacterial diversity generated by primer pair 338f/806r was significantly impacted by mean annual precipitation, soil total phosphorus (TP), soil total nitrogen (TN), and soil available phosphorus (AVP), while the soil bacterial diversity generated by primer pair 520f/802r showed no significant correlations with most of these environmental factors. Multiple regression models indicated that soil pH and soil organic carbon (SOC) shaped the soil bacterial community structure on the Loess Plateau regardless of what primer pair was used. Climatic conditions mainly affected the diversity of rare bacteria. Abundant bacteria are more sensitive than rare bacteria to environmental changes. Very little of the variation in the rare bacterial community was explained by environmental factors or geographic distance, suggesting that the communities of rare bacteria are unpredictable. The distributions of the abundant taxa were mainly determined by variations in environmental factors.

## 1. Introduction

Soil harbors many highly taxonomically and metabolically diverse microorganisms, which drive multiple ecological functions, such as nutrient cycling, climate regulation, carbon sequestration, soil health, and ecosystem stability. To date, most studies associated with soil microbial communities have focused on highly abundant species rather than rare species. Despite having low abundance, communities of rare microorganisms exhibit high genetic and functional diversity and may regulate biogeochemical cycling and ecosystem stability [1]. For example, low-abundance microorganisms have greater metabolic capacity and grow faster in soils [1]. Rare microorganisms reduce sulfate content in peat soils [2]. In a long-term fertilization experiment, a rare soil bacterial community, rather than a dominant soil bacterial community, controlled multiple ecosystem functions [3]. Li et al. (2019) observed distinct changes in rare and abundant bacteria in response to humic acid amendment [4]. Similarly, a previous study reported that different patterns were observed between rare and abundant taxa in oil-contaminated soils [5]. These results highlighted the key roles and high sensitivity of rare bacterial communities in soils in response to environmental conditions. Recently, some studies focused on the biogeographic distributions and driving forces of abundant and rare microbes in agricultural soils [6,7], grassland soils [8] and temperate deserts [9]. These results showed that different patterns for abundant and rare microbes were detected in different ecosystems. In temperate deserts, similar abiotic and biotic drivers shaped the distributions of abundant and rare bacteria [9], while in agricultural soils, soil pH and mean annual temperature regulate the assembly of abundant and rare bacteria [7]. Hence, clarifying the geographic patterns of rare bacteria in arid and semiarid areas may provide new evidence for the mechanism of soil microbial community assembly in response to different land uses.

The recent and rapid development of high-throughput sequencing has allowed for the identification of rare microbial communities [10]. This method has been used for microbial identification in diverse environmental conditions, such as oil-contaminated soils [5], activated sludge [11], and lakes and reservoirs [12]. However, the use of different primer sets targeting different genes may lead to different microbial community diversity and composition values. Generally, primers designed to target 16S rDNA are species-specific [13]. Therefore, the targeted V region and related primer pairs should be carefully selected to ensure that the relevant microbial groups are covered and that the microbiome is accurately displayed in the analysis [14,15,16]. Different regions of the bacterial 16S rRNA gene have evolved at different rates, so the results of these analyses may vary with the region sequenced [16,17,18,19].

Land use, as an important consequence of human activity, has caused many variations in aboveground and belowground ecosystems. For example, modern agricultural practices, which are based on industrially produced mineral fertilizers, have been shown to reduce soil carbon content, N fixation ability, and plant diversity [20,21]. The conversion of paddy fields to orchard farms causes losses in soil biodiversity and changes in soil microbial community structures [22]. The abundance and richness of soil microarthropods significantly increased after the conversion of native steppe to farmland [23]. These results show that land use plays a central role in regulating soil biodiversity and plant diversity. However, the variations in and underlying drivers of abundant and rare microbial communities in soil in arid and semiarid areas are not well understood.

In arid and semiarid ecosystems, water availability can be a primary factor in regulating biological resources, such as soil fertility, plant biomass, and soil organisms [24]. Climatic features was shown to alter microbial diversity and abundance globally [25]. Therefore, in arid or semiarid areas, the biogeography of rare and abundant bacterial species may be different from that in areas with higher precipitation. In this study, we used two primer sets to assess the variations in and underlying mechanisms of the diversity and community structure of the rare and abundant bacteria in soils on the Loess Plateau along a latitudinal gradient. The land uses in this area include forest, grassland, cropland, orchard, and shrubland. Soil bacterial diversity and community structure were analyzed using 16S rRNA gene sequencing with two primer pairs: 338f/806r, which targets the V3–V4 region, and 520f/802r, which targets the V4 region. We aimed to address the following questions. (1) Do rare and abundant taxa respond similarly to environmental factors? (2) Of local environmental factors and geographic distance, which variables controlled the differences in the geography of the rare and abundant soil bacterial communities?

## 2. Methods and Materials

### 2.1. Site Description and Sampling

This study was conducted on the Loess Plateau, which represents one of most important arid–semiarid areas in China. We established 24 field sites for sampling on the Loess Plateau, spanning ~400 km from north to south [26]. These sites contain five land uses (forest, grassland, shrubland, orchard, and cropland) and are distant from human settlements to avoid the influences of human activities. Loessial soils were the main soil type according to the Genetic Soil Classification of China [27]. All the sites had a relatively low mean annual precipitation (MAP, 372–585 mm) and mean annual temperature (MAT, 7.61–13.08 °C).

We collected field soil samples in July (summer) 2016. First, three plots (20 m × 20 m) were established in each site. In total, 20 surface soil cores were collected from three plots using an auger (5 cm diameter) at 0–10 cm soil depth to obtain a representative sample for one site. All the soil cores from one site were sieved through a 2 mm mesh. During this sieving process, roots, plant litter, and stones were removed by hand. After sieving, the soil samples were stored according to the requirements of the different analyses. Approximately 10 g of soil was stored in a 10 mL centrifuge tube at −80 °C for DNA extraction; the remaining sieved soils were air-dried at room temperature (~25 °C) in the dark and used to analyze the soil properties.

### 2.2. Climate and Edaphic Characteristics

To obtain the specific climate data at each sampling site, we used spatial interpolation in ArcGIS 10.0 using the MAT and MAP datasets from 154 weather stations covering the whole Loess Plateau. After spatial interpolation, we retrieved the specific climate data for the latitude and longitude of each sampling site. This analysis has been widely used in obtaining climate data. The basic soil characteristics were determined previously [26] by standard methods described elsewhere [26,28,29]. The data are listed in the supporting information (Table S1).

### 2.3. DNA Extraction

A PowerSoil kit (MoBio Laboratories, Carlsbad, CA, USA) was used to extract DNA from 0.5 g of frozen soil according to the manufacturer’s instructions. The gDNA samples described in Liu et al. (2018) [26] were used in the current study.

### 2.4. 16S rRNA Gene Amplification and Sequencing

Two primer pairs targeting different hypervariable regions were used for amplification. Primers 338F (5′-ACTCCTACGGGAGGCAGCA-3′) and 806R (5′-GGACTACHVGGGTWTCTAAT-3′) targeted the V3–V4 regions of the 16S rRNA genes [30], and primers 520F (5′-CCATCTCATCCCTGCGTGTCTCCGAC-3′) and 802R (5′-CCTCTCTATGGGCAGTCGGTGAT-3′) targeted the V4 region of 16S rRNA genes [31]. The amplification conditions were as follows: 50 s at 94 °C, 30 s at 40 °C, 35 cycles of 60 s at 72 °C, followed by 5 min at 72 °C. Sequencing was performed on an Illumina MiSeq platform with chemistry version 3 (2 × 300 bp) at Shanghai Personal Biotechnology Co., Ltd. (Shanghai, China).

### 2.5. Processing of the Sequencing Data

The raw sequences were processed in QIIME (v 1.17), including quality control, operational taxonomic unit (OTU) clustering, and taxonomic assignment. Short sequences (<20 nucleotides) were first removed and then chimeric sequences were identified and removed. After quality control, the sequences were clustered into operational taxonomic units (OTUs) at 97% similarity using UPARSE [32] (version 7.1 http://drive5.com/uparse/). Taxonomy was assigned using the Ribosomal Database Project (RDP) classifier. In order to compare the differences of different samples with the two pairs of primers, we used the same sequence number (9640 sequences/sample) to obtain bacterial diversity and community. Rare bacteria were defined as OTUs with a lower relative abundance (<0.05%), and abundant bacteria were defined as OTUs with a higher relative abundance (1%) [12].

### 2.6. Statistical Analysis

ANOVA was performed using SPSS 20.0 to assess the differences in bacterial diversity in soils under different land uses. Correlation analysis was performed in R 3.5.3 to evaluate the associations between community diversity and environmental factors [33]. The differences in the bacterial community structures were visualized with a nonmetric multidimensional scaling (NMDS) plot. The relationships between the bacterial community and environmental factors were determined by a Mantel test in R 3.5.3, which individually tested the effects of each environmental factor in regulating the bacterial community structure. Multiple regression models were conducted to further distinguish the contributions of each factor to shaping the bacterial diversity and community structure. The Akaike information criterion (AIC) scores were used to assess the goodness of fit for the multiple regression models [34], and the relative importance (RI) was calculated to quantify the effects of each factor in regulating microbial community and diversity [35,36]. Variance partitioning analysis (VPA) was performed in R 3.5.3 to distinguish the relative importance of local environmental factors and geographic distance in explaining the variations in the bacterial community structure.

## 3. Results

### 3.1. The Distributions of the Soil Bacterial Diversity

**Figure 1:**
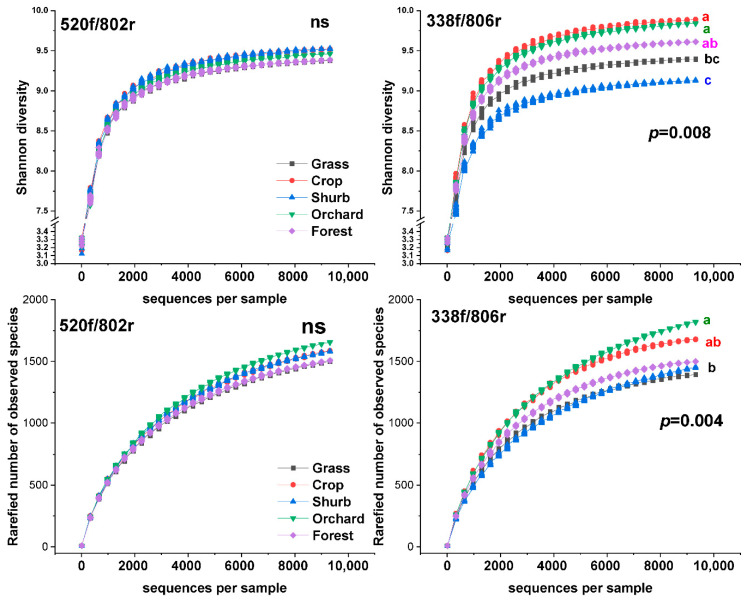
The Shannon diversity index and richness of soil bacteria in different land uses on the Loess Plateau. Primer pair 520f/802r targeted the V4 region of the 16S rRNA gene; primer pair 338f/806r targeted the V3–V4 region of the 16S rRNA gene. Ns, no significant difference.

The primer pairs had no significant effects on detecting soil bacterial diversity. The two primer pairs showed different changes in bacterial diversity in the soils among the five land uses (Figure 1). The variations in the observed number of OTUs and Shannon diversity index were more evident for the 338f/806r primer pair than the 520f/802r primer pair, suggesting that the 338f/806r pair can clearly reflect the variations in the bacterial diversity. For the 338f/806r primers, the cropland soils had the highest bacterial Shannon diversity index, followed by the orchard, forest, grassland, and shrubland soils. Similarly, the observed number of OTUs was highest in the orchard soils, followed by the cropland, forest, shrub, and grassland soils according to the results based on primer pair 338f/806r.

The Pearson correlation analysis of the samples obtained with the 520f/802r primer pair showed that most environmental factors (except soil NO3N) had no significant effects on the soil Shannon diversity index. For the 338f/806r primers, the Shannon diversity index was significantly associated with MAP (r = 0.723), MAT (r = 0.529), available phosphorus (AVP) (r = 0.48), total phosphorus (TP) (r = 0.57), and latitude (r = −0.62) (Figure S1). The multiple regression models revealed that MAP contributed the most to variations in the bacterial Shannon diversity index, with a relative importance of 0.70, suggesting the overwhelming effects of MAP in regulating bacterial diversity (Table 1).

### 3.2. The Distributions of the Soil Bacterial Community Compositions

**Figure 2:**
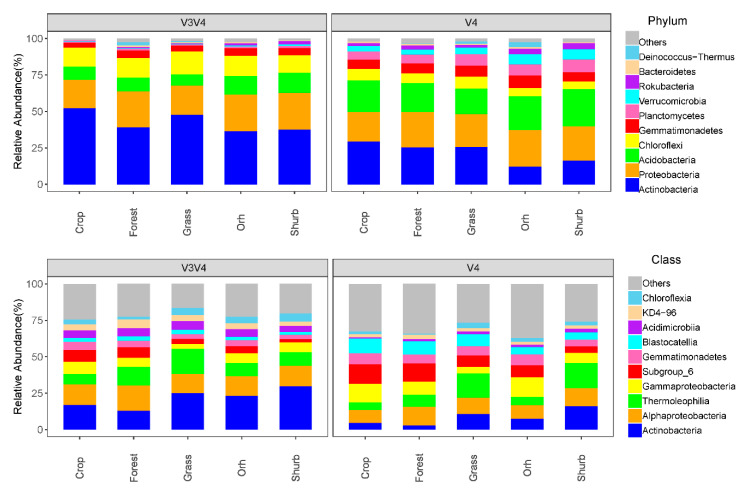
The community compositions of soil bacteria in different land uses on the Loess Plateau. The top panel represents the distributions of the soil bacterial community at the phylum level; the bottom panel represents the distributions of the soil bacterial community at the class level. Orh refers to orchards. Primer pair 520f/802r targeted the V4 region of the 16S rRNA gene; primer pair 338f/806r targeted the V3–V4 region of the 16S rRNA gene.

The two primer pairs detected similar bacterial community taxa at different taxonomic levels. Actinobacteria, Proteobacteria, Acidobacteria, Chloroflexi, Gemmatimonadetes, Planctomycetes, and Verrucomicrobia were the most abundant phyla detected by the two primer pairs (Figure 2). The primer pair 338f/806r detected more Actinobacteria (42.1%) and Chloroflexi (13.8%) than did the primer pair 520f/802r (21.6% and 6.8%, respectively). The primer pair 520f/802r detected more Acidobacteria, Gemmatimonadetes, Planctomycetes, and Verrucomicrobia than did the primer pair 338f/806r. The two primer sets detected similar relative abundances of Proteobacteria (23.5% for primer pair 520f/802r and 23.1% for primer pair 338f/806r).

For the two primer pairs, the effects of land use on the soil bacterial community compositions were different. The results from both primer pairs revealed that the shrubland and grassland soils had the highest relative abundance of Actinobacteria, while the cropland and forest soils had the lowest relative abundance of Actinobacteria. However, the variations in abundance were more evident for primer pair 520f/802r. There were no significant differences in Proteobacteria among the five land uses based on the samples obtained with the 520f/802r primer pair; however, Proteobacteria were significantly more abundant in the cropland and forest soils than in the grass soils according to the sequencing data obtained using primer pair 338f/806r. Primer pair 338f/806r showed more obvious variations than did the primer pair 520f/802r in Acidobacteria. The shrubland soils had the lowest abundance of Acidobacteria, while the cropland and forest soils had relatively more Acidobacteria. There was no significant difference in the relative abundance of Chloroflexi between the different land uses based on the data obtained using either primer pair.

### 3.3. The Variations in the Soil Bacterial Community Structure

**Figure 3:**
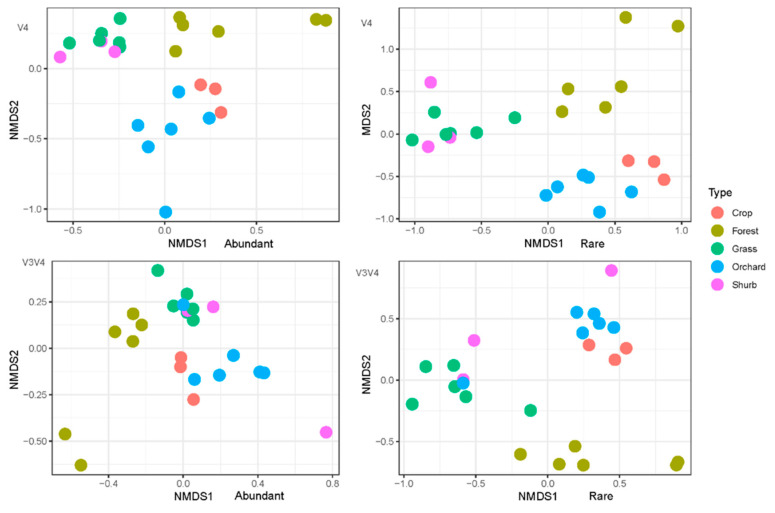
The community structure of abundant and rare soil bacteria in different land uses. Primer pair 520f/802r targeted the V4 region of the 16S rRNA gene; primer pair 338f/806r targeted the V3–V4 region of the 16S rRNA gene.

The NMDS plot showed significant differences between the primer pairs rather than the land uses. The different land use samples were clustered together for the two primer pairs, suggesting that land use had strong effects on shaping the bacterial community structure in soils (Figure S2). MAT, MAP, latitude, pH, soil organic carbon (SOC), and total nitrogen (TN) were significantly associated with NMDS1 (Figure S3). Abundant and rare bacterial groups showed similar patterns among the different land uses (Figure 3). The grassland and shrubland samples had similar abundant and rare bacterial community structures, and the orchard and cropland samples clustered together. For the two primer pairs, the Mantel test showed that MAP, MAT, SOC, TN, and pH were significant factors influencing bacterial community structure (Figure S4). The multiple regression models showed that pH and SOC were the main driving forces of soil bacterial community structure based on the samples obtained with these two primer pairs. For primer pair 338f/806r, pH, SOC, electrical conductivity (EC), and AVP were the main factors influencing the soil bacterial community structure, with relative importance values of 0.46, 0.41, 0.07, and 0.06, respectively (Table 2), while for primer pair 520f/802r, pH and SOC significantly affected the soil bacterial community structure, with relative importance values of 0.46 and 0.54, respectively (Table 3).

**Figure 4:**
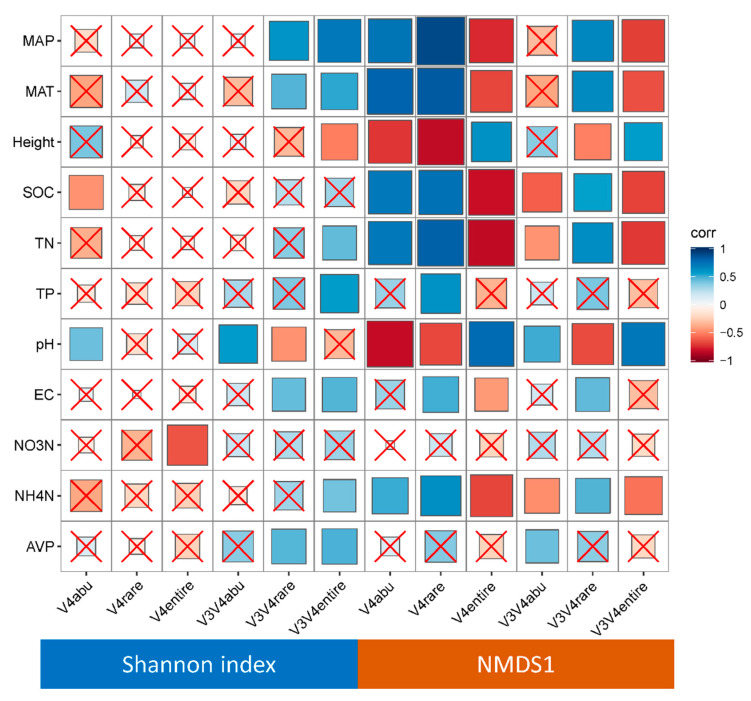
The associations between the diversity (Shannon index) and community structure (nonmetric multidimensional scaling 1 (NMDS1)) of abundant and rare soil bacteria in different land uses. Primer pair 520f/802r targeted the V4 region of the 16S rRNA gene; primer pair 338f/806r targeted the V3–V4 region of the 16S rRNA gene. The red crosses indicate nonsignificant correlations, and the size of the squares suggest higher Pearson coefficients. MAP, annual mean precipitation; MAT, annual mean temperature; SOC, soil organic carbon; EC, electrical conductivity; AVP, soil available phosphorus; TN, total nitrogen; TP, total phosphorous; NO3N, nitrate nitrogen; NH4N, ammonia nitrogen; abu, abundant bacterial taxa; rare, rare bacterial taxa; entire, the entire bacterial community.

For primer pair 520f/802r, the associations between the abundant bacterial community and environmental factors were similar to those between the rare bacterial community and environmental factors (Figure 4). However, for primer pair 338f/806r, the association of the environmental factors and abundant bacterial community was opposite that of the environmental factors and rare bacterial community. For example, the abundant bacterial community was positively associated with soil pH, while the rare bacterial community was negatively associated with soil pH.

### 3.4. Geographic Distance and Environmental Factors Regulate Bacterial Community Structure

**Figure 5:**
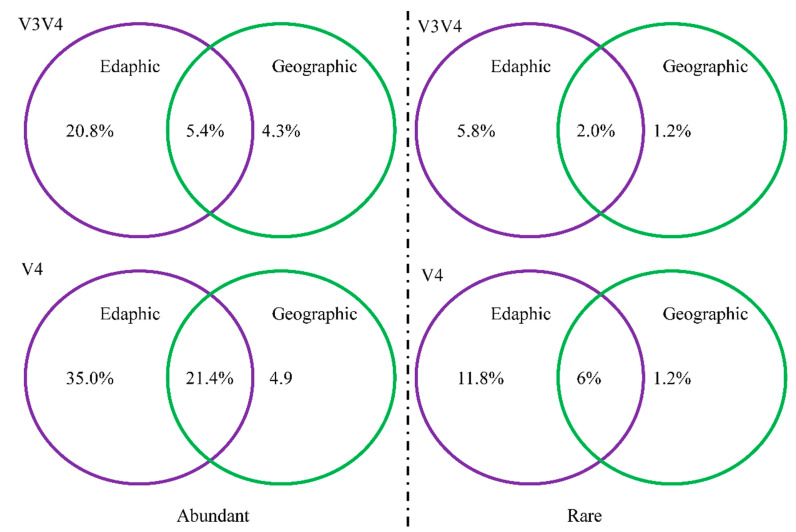
The variation in the community structure of abundant and rare soil bacteria explained by edaphic and geographic factors. Primer pair 520f/802r targeted the V4 region of the 16S rRNA gene; primer pair 338f/806r targeted the V3–V4 region of the 16S rRNA gene.

For both primer pairs, variance partition analysis showed that environmental factors contributed more than geographic distance to explaining the variation in the abundant and rare bacteria. Specifically, for primer pair 338f/806r, a total of 30.5% of the variation in the abundant bacterial community could be explained, with 20.8% explained by environmental factors and 4.3% explained by geographic distance. For primer pair 520f/802r, altogether, the measured variables explained 51% of the variation in the abundant bacterial community; environmental factors and geographic distance independently explained 35% and 4.9% of this variation, respectively. For the rare bacteria, geographic distance contributed little (1.2%) to shaping the community structure based on either primer pair. Environmental factors explained 5.8% (338f/806r) and 11.8% (520f/802r) of the variation in the rare bacterial community structure (Figure 5).

## 4. Discussion

### 4.1. Land Use Affected the Soil Microbial Diversity

We used two bacteria-specific primers to evaluate the bacterial community diversity in soils and their controlling factors under different land uses in an arid–semiarid area. The results demonstrated that primer pair 338f/806r generated a higher number of OTUs and Shannon diversity index than those of primer pair 520f/802r, but the difference was not significant. The cropland and orchard soils had the highest bacterial Shannon diversity indexes, which were significantly different from that of the grassland, forest, and shrubland soils. Land use has been reported as an important driver in impacting soil bacterial diversity [7]. The differences in the Shannon diversity index generated by the two primer pairs may indicate the possibility of drawing comparisons among previous studies that used different primer pairs [14,15,16].

In arable land, the input of mineral fertilizer could stimulate the growth and reproduction of soil microbes, resulting in increases in soil microbial diversity, such as in croplands and orchards. High nutrient availability would promote the growth of soil copiotrophs [37]. Compared with arable soils, in the natural vegetation soils, we found lower soil bacterial diversity. This relatively low bacterial diversity in the grassland might be due to the lower input of nutrients than in other soils. Cropland and orchard soils had relatively more nutrients, such as TN and AVP contents, which may promote the growth of some taxa and enhance the diversity. Continuous crop cultivation increased soil bacterial diversity via soil nutrient supply [38]. In our study, soil AVP mainly affected rare bacterial diversity, whereas the abundant bacterial diversity has no significant associations with AVP. These results highlighted that soil nutrients would impact the diversity of soil bacteria, as previous studies reported that soil properties caused by land use type shaped soil bacterial community on the Loess Plateau [39,40]. Another potential factor is precipitation, which controls bacterial diversity, especially among rare bacteria. A global study showed that soil bacteria diversity was decreased as aridity increased [41]. We found that rare bacterial diversity (V4) significantly increased with elevated MAP. The abundant bacterial diversity generated by the two primer sets was stable with lower versions in changing climatic regimes. In arid–semiarid regions, precipitation might be the main limiting factor mediating soil microbial activity [42]. The increase in aridity decreased the content of soil organic C and total N but increased the content of inorganic P [43]. A previous study suggested that rare communities comprised more functional taxa in regulating the nutrient cycling [3] and were more sensitive to changing climatic regimes. In this study, the positive associations between bacterial diversity and MAP highlighted the critical role of precipitation in altering soil rare bacterial diversity.

### 4.2. Land Use Affected the Soil Microbial Community Compositions

Among the sequences obtained by the two primer pairs, different dominant OTUs were found, which led to the differences in the relative abundances of the different bacterial phyla. Primer pair 520f/802r identified more Acidobacteria, Gemmatimonadetes, Planctomycetes, and Verrucomicrobia, while primer pair 338f/806r identified more Actinobacteria and Chloroflexi. Although the differences in the relative abundances of these phyla were significant, the bacterial community structure was similar among the different land uses. The results from the two primer pairs demonstrated that Proteobacteria, Actinobacteria, Acidobacteria Chloroflexi, and Acidobacteria were the dominant phyla (Figure 2); these phyla have been widely detected in various soils and sediments [44,45,46,47], but their relative abundances varied dramatically according to land use and soil conditions. The variations in the abundance of these bacterial taxa might be attributed to pH, nutrients, and climate conditions.

The main bacterial phyla in the different land uses showed similar trends for both primer pairs. For example, the relative abundances of Actinobacteria in the cropland, orchard, and forest soils were similar and were significantly lower than those in the grassland or shrubland soils. The grassland and shrubland soils had the lowest Proteobacteria abundance, which may be due to these areas having the lowest soil organic carbon. The variations in the soil bacterial abundance at the phylum level might be associated with changes in resource availability [37]. Previous studies have shown that soil organic carbon was the main driver of the composition of soil bacterial taxa [37,48,49]. In this study, SOC was significantly related to the main bacterial taxa (Actinobacteria, Proteobacteria, Verrucomicrobia, and Acidobacteria). In agreement with a previous study reported by Zeng et al. (2019), there were significant correlations between SOC and the relative abundances of Actinobacteria, Verrucomicrobia, and Acidobacteria in the loessial soils [50]. In contrast, we found a negative relationship between the relative abundance of Actinobacteria and SOC, which was opposite to the findings of other studies [49]. Many studies have reported that Actinobacteria was categorized as a copiotrophic group and favored soils high in SOC [37,51]; however, in our study, Actinobacteria were more abundant in soils with lower SOC contents, and a similar study reported that Actinobacteria was enriched in soils low in SOC [50]. The different responses of Actinobacteria to SOC content may be explained by their diverse life strategies. Actinobacteria have different life strategies depending on nutrient conditions [52]. For example, Trivedi et al. (2015) reported that Actinobacteria and δ-Proteobacteria are categorized as oligotrophic bacteria (k-strategists), which can degrade relatively recalcitrant forms of C and are dominant in nutrient poor conditions [52]. The relative abundance of Actinobacteria was much higher in soils with relatively low SOC contents, indicating that Actinobacteria are oligotrophic on the Loess Plateau. This finding suggests that Actinobacteria are widely distributed in soils globally, but their life strategies or functions might differ [53,54] under different soil conditions.

Another interesting result was that Acidobacteria were more abundant in soils with higher SOC contents; this finding was inconsistent with those of other studies [37]. Fierer et al. (2007) proposed that Acidobacteria have an oligotrophic life strategy and might be more competitive in soils with lower SOC contents [37]; however, this was not consistent with our findings in this study. In the present study, Acidobacteria were more abundant in soils with higher SOC contents, suggesting that they are copiotrophs, which is in line with the study reported in the Loess Plateau [55]. Most members of the Proteobacteria (α, β, and γ Proteobacteria) are copiotrophic and can produce enzymes to degrade labile C and grow faster in high-nutrient conditions. This was confirmed by the positive association between the relative abundance of Proteobacteria and SOC content (Table S2). Therefore, the abundance of Proteobacteria may be enriched in forest soils because of the higher SOC content. In forest soils, there is higher resource availability due to plant litter and roots, which enhances the growth of copiotrophic taxa, such as Proteobacteria and Acidobacteria. Taken together, although the dominant phyla were similar to those in other studies, the life strategies of these phyla were obviously different in this study. Therefore, future studies should focus on the functions of the dominant taxa in soils globally.

### 4.3. The Role of Environmental Factors in Shaping Soil Bacterial Community Structure

The significant correlations between geographic distance and the dissimilarity of soil bacterial communities demonstrate that dispersal limitation is also an important factor affecting the soil bacterial communities generated by the two primer pairs. Some studies have reported that the distributions of soil bacterial communities were controlled by geographic distance rather than local environments [56]; others hold the point of view that environmental forces have a greater influence than geographic patterns on soil bacteria [50,57,58,59]. The VPA showed that in this study, environmental variables (which explained 19% of the variation for primer 338f/806r and 23% for primer 520f/802r) played a more important role than geographic distance (which explained 4% of the variation for primer 338f/806r and 7% for primer 520f/802r) in shaping soil bacterial spatial patterns. Similarly, Liu et al. (2014) reported that environmental variables (37.52%) explained more of the variation in the bacterial community structure than did geographic distance (14.75%) in the northwest of China (spanning 800 km) [49].

Similar geographic patterns of abundant and rare bacteria were observed based on both primer sets. The edaphic properties explained more of the variation in the bacterial distributions than did geographic distance for the abundant and rare bacterial communities. The VPA showed that the edaphic properties tended to explain more of the variation in the bacterial community for the sequences obtained from primer pair 520f/802r (targeting the V4 region) (35.0%) than for those obtained from primer pair 338f/806r (targeting the V3–V4 region) (20.8%). Remarkably, spatial and environmental variables explained a relatively low amount of the variation (9% for primer pair 338f/806r; 19% for primer pair 338f/806r) in the rare bacterial community, and the relative contribution of spatial factors was very low (1.2%). Similarly, spatial factors had no significant effects on soil rare microbial communities in agricultural soils (intervals of 18 to 3689 km) [33]. In contrast, Wang et al. (2020) reported that spatial factors explained a higher proportion (8%) of soil rare bacterial compositions in temperate deserts (in a 2500 km regional transect) [9]. In the Tibetan Plateau, geospatial distance (22.1%) contributed more to variations in the abundant community composition than local factors (11.3%) (in a 1200 km transect) [8]. This disagreement might be attributed to the small scales used and effects of different ecosystems and environmental factors. The results of our study suggest that local environmental processes control the shifts in the abundant and rare bacteria. This is line with a previous study, which found that edaphic factors determined the alterations in the community structure of abundant microorganisms [51].

Among the main environmental factors, soil pH emerged as the most important factor driving the composition of abundant and rare bacterial communities in soil on the Loess Plateau. It is widely accepted that soil pH determines the bacterial community composition in various soils. Previous studies have confirmed that soil pH controls the distribution of the soil bacterial community in North American soils [60], British soils (2331 km^2^ squares across the UK) [61], black soils (spanning 800 km) [49], loessial soils (spanning 400 km) [50], Chinese forest soils (18.70° N–51.53° N) [59], agricultural soils (intervals of 18 to 3689 km) [7], and Arctic soils [58]. In our study, although the soil type was similar among the study sites, the soil pH ranged from 6.14 to 9.12, with a large variation, and most (92%) of the soil samples were alkaline, which is different from previous studies [48,49,58,59] in which the soil pH ranged from 4 to 7. Similarly, Jiao et al. (2020) reported that soil pH regulates the assembly of the abundant microbial communities with a wide pH (~4–~9) range in agro-ecosystems [7]. Xia et al. (2016) reported that Acidobacteria was not correlated with soil pH, while the relative abundance of Chloroflexi, Betaproteobacteria, Deltaproteobacteria, Gemmatimonadetes, Nitrospirae, and Bacteroidetes was positively correlated with soil pH (4.13–7.54) [59]. We also observed significant correlations between soil pH and the relative abundances of Actinobacteria, Proteobacteria, and Acidobacteria, which accounted for 66% and 76% of the taxa identified in the samples obtained with the primer pairs 520f/802r and 338f/806r, respectively. In contrast, significant correlations between the relative abundance of Chloroflexi and soil pH were observed for only primer pair 520f/802r (not 338f/806r), while for Planctomycetes, significant correlations between relative abundance and soil pH were observed for only primer pair 338f/806r. The differences between the primer pairs may lead to different correlations between soil phyla and environmental factors.

## 5. Conclusions

This study provides strong evidence that the primer pairs have significant effects on the bacterial α-diversity values of soil rather than on its bacterial community structure. Although the different primer sets generated different dominant taxa, the bacterial community structure was still similar. For both primer pairs, similar dominant bacterial phyla were detected, but with different relative abundances. SOC content and its association with soil pH have been considered the main drivers of the bacterial community structure in soils on the Loess Plateau. Similar spatial patterns were observed in the rare and abundant bacterial communities. Compared with local environmental factors, geographic distances had limited impacts on the abundant and rare bacterial communities in the soils sampled. Compared with the abundant bacteria, the rare bacteria in the soils were less strongly associated with environmental conditions. Our results provide new insights into the geographic distributions and controlling factors of rare bacterial communities in arid and semiarid regions. Future studies need to focus on the functions of rare and abundant species, which would provide a better understanding of the roles of microbes in maintaining ecosystem functioning and services at the local and global scales.

## Figures and Tables

**Table 1:** The impacts of environmental factors on the soil bacterial diversity of sequences obtained using the 338f/806r primer set targeting the V3–V4 region.

V3–V4 (Shannon Diversity)	Estimate	t	*p*	RI
(Intercept)	6.727	14.906	<0.0001	
MAP	0.005	5.803	<0.0001	0.7029
NH4N	0.111	2.431	0.0246	0.1461
SOC	−0.063	−3.344	0.0032	0.1510

MAP, mean annual precipitation; NH4N, ammonia nitrogen; SOC, soil organic carbon; RI, relative importance calculated from the multiple regression models.

**Table 2:** The impacts of environmental factors on the soil bacterial community structure of the sequences obtained using the 338f/806r primer set targeting the V3–V4 region.

V3–V4 (NMDS1)	Estimate	t	*p*	RI
(Intercept)	−0.645	−1.373	0.186	
pH	0.171	3.4	0.003	0.4561
SOC	−0.022	−3.208	0.005	0.4103
EC	0.000	2.245	0.037	0.0742
AVP	−0.005	−2.333	0.031	0.0594

RI, relative importance calculated from the multiple regression models; SOC, soil organic carbon; EC, electrical conductivity; AVP, soil available phosphorus.

**Table 3:** The impacts of environmental factors on the soil bacterial community structure of the sequences obtained using the 520f/802r primer set targeting the V4 region.

V4 (NMDS1)	Estimate	t	*p*	RI
(Intercept)	−1.632	−4.431	0.00023	
pH	0.143	3.598	0.00169	0.4598
SOC	−0.025	−4.432	0.00023	0.5402

RI, relative importance calculated from the multiple regression models; SOC, soil organic carbon.

## Supplementary Materials

The following are available online at https://www.mdpi.com/2076-2607/9/1/139/s1, Figure S1: The associations between bacterial diversity and environmental factors using linear regressions, Figure S2: The community structure under different land uses using NMDS plots, Figure S3: The associations between bacterial community structure and environmental factors using linear regressions, Figure S4: The associations between community structure and environmental factors using mantel test. V3V4 means the bacterial community structure tested by the primer pairs of 338f/806r targeting V3–V4 region; and V4 means the bacterial community structure tested by the primer pairs of 520f/802r targeting V4 region, Table S1: The characteristics of different sampling sites on the Loess Plateau, Table S2: The associations between environmental factors and main bacterial phyla.
