# Supplementary material for: Identifying the Biogeographic Patterns of Rare and Abundant Bacterial Communities Using Different Primer Sets on the Loess Plateau
Source: Microorganisms. 2021 Jan 9;9(1):139. doi: 10.3390/microorganisms9010139 (PMC7827256; doi:10.3390/microorganisms9010139)
Supplement: Supplementary file 1 [file microorganisms-09-00139-s001.pdf]

Supporting information

Identifying the Biogeographic Patterns of Rare and Abundant Bacterial Communities Using Different Primer Sets on the Loess Plateau

Quanchao Zeng[a,b] and Shaoshan An[b*]

[a] College of Resources and Environment, Huazhong Agricultural University, Wuhan 430070, PR China
[b] State Key Laboratory of Soil Erosion and Dryland Farming on the Loess Plateau, Institute of Soil and Water Conservation, Northwest A&F University
[*] Corresponding author: Shaoshan An, E-mail: shan@ms.iswc.ac.cn

Tables: 2

Figures: 3

Table S1 The characteristics of different sampling sites on the Loess Plateau[1]

Site	MAP	MAT	Height (m)	SOC (g/hg)	TN (g/hg)	TP (g/hg)	pH	EC (μS/cm)	NH4N (mg/hg)	AVP (mg/hg)	C/N	Shannon-V4	Shannon-V3V4
ASA	474.5	10.0	1273	3.35	0.32	0.53	8.90	91.67	7.32	0.78	10.63	9.30	9.52
ASB	484.4	10.0	1298	4.17	0.33	0.49	8.96	102.67	6.90	1.63	12.57	9.50	9.41
ASC	484.4	10.0	1335	8.03	0.47	0.56	8.79	134.00	7.95	2.08	17.24	9.83	9.61
GZA	572.7	12.4	656	9.89	1.11	0.93	8.54	152.00	8.23	11.82	8.93	9.62	9.82
GZB	560.6	12.9	549	9.95	1.02	0.95	8.43	297.67	8.31	17.27	9.74	9.53	9.91
GZC	535.4	13.4	415	12.49	0.94	1.18	8.55	263.00	9.85	26.75	13.26	9.42	9.95
JBA	369.4	9.3	1563	2.91	0.27	0.32	8.91	91.67	5.86	0.73	10.64	9.99	8.55
JBB	371.7	9.3	1633	2.61	0.29	0.26	9.02	66.33	7.53	0.55	8.93	9.17	9.68
JBC	374.2	9.3	1572	4.54	0.45	0.43	8.92	72.33	8.13	0.42	10.19	9.44	9.18
LCA	559.8	10.9	1206	8.09	0.97	0.96	8.32	611.33	7.93	72.07	8.36	9.98	9.86
LCB	558.5	10.9	1220	8.61	1.09	1.05	8.31	1076.00	8.64	62.87	7.89	9.59	9.76
LCC	561.0	10.7	1138	6.88	0.77	0.68	8.45	203.67	8.33	19.69	8.99	9.99	10.15
LDWA	412.1	9.5	1350	5.05	0.45	0.46	8.82	87.67	7.00	0.62	11.11	8.98	9.09
LDWB	409.8	9.5	1420	2.66	0.18	0.47	9.11	75.33	6.69	0.49	14.70	9.38	9.43
LDWC	406.5	9.5	1403	4.09	0.27	0.49	9.12	74.00	6.50	0.52	14.98	9.33	9.36
QLA	560.5	13.6	708	15.03	1.28	0.56	6.80	264.67	10.70	3.13	11.72	9.10	9.60
QLB	577.6	12.9	754	21.63	1.99	0.59	7.65	603.00	14.75	4.70	10.85	9.77	9.75
QLC	568.2	13.5	822	14.87	1.31	0.54	6.14	385.00	9.02	3.27	11.39	9.31	9.77
WBA	547.7	12.9	421	10.00	1.01	1.10	7.81	472.00	8.09	24.70	9.93	9.82	9.46
WBB	557.8	12.7	397	9.92	1.00	1.38	8.42	731.00	8.89	51.27	9.95	9.48	9.91
WBC	547.4	10.5	1180	9.06	1.20	1.52	7.78	828.00	11.30	87.27	7.52	7.91	9.94
ZWLA	546.6	10.3	1185	15.62	1.36	0.54	8.38	371.33	10.28	3.68	11.47	9.58	9.66
ZWLB	537.7	10.1	1025	10.59	0.95	0.47	8.87	90.67	8.76	2.29	11.11	9.62	9.76
ZWLC	519.7	9.8	1151	18.76	1.41	0.58	8.73	99.67	10.57	4.00	13.27	8.95	9.18

Table S2 The associations between environmental factors and main bacterial phyla

Variables	Actinobacteria		Proteobacteria		Acidobacteria		Chloroflexi		Planctomycetes		Verrucomicrobia		Nitrospirae		Firmicutes	
	V3V4	V4	V3V4	V4	V3V4	V4	V3V4	V4	V3V4	V4	V3V4	V4	V3V4	V4	V3V4	V4
MAP	-0.617	-0.925	0.549	0.630	0.680	0.451	0.103	-0.269	0.562	0.471	0.328	0.335	0.508	0.257	-0.297	-0.349
MAT	-0.567	-0.785	0.508	0.434	0.608	0.589	0.047	-0.297	0.443	0.219	0.363	0.432	0.711	0.084	-0.327	-0.308
Height	0.503	0.793	-0.397	-0.324	-0.600	-0.620	-0.094	0.304	-0.491	-0.397	-0.202	-0.298	-0.558	-0.078	0.290	0.333
SOC	-0.569	-0.738	0.552	0.419	0.737	0.558	-0.298	-0.502	0.674	0.280	0.581	0.508	0.326	0.086	-0.111	-0.258
TN	-0.635	-0.807	0.585	0.566	0.696	0.387	-0.168	-0.449	0.673	0.386	0.463	0.387	0.417	0.147	-0.084	-0.265
TP	-0.389	-0.614	0.234	0.368	0.238	-0.041	0.376	-0.191	0.385	0.628	-0.291	-0.202	0.408	-0.078	-0.016	-0.240
pH	0.566	0.664	-0.538	-0.401	-0.562	-0.301	0.006	0.308	-0.228	-0.096	-0.687	-0.687	-0.587	0.058	0.278	0.190
EC	-0.336	-0.561	0.281	0.563	0.134	-0.168	0.161	-0.190	0.197	0.510	-0.088	-0.072	0.225	-0.105	0.012	-0.131
NH4N	-0.472	-0.575	0.477	0.413	0.652	0.310	-0.278	-0.479	0.695	0.176	0.341	0.238	0.269	-0.006	-0.113	-0.267
AVP	-0.298	-0.424	0.194	0.451	-0.034	-0.404	0.449	-0.069	0.179	0.597	-0.323	-0.272	0.221	-0.097	0.149	-0.090
C.N	0.324	0.237	-0.213	-0.399	0.028	0.422	-0.367	-0.140	-0.058	-0.349	0.144	0.185	-0.231	-0.114	-0.271	0.010
Longitude	0.529	0.351	-0.468	-0.247	-0.556	-0.220	0.265	0.441	-0.669	0.140	-0.363	-0.244	-0.694	-0.161	0.094	0.108
Latitude	0.682	0.892	-0.601	-0.538	-0.720	-0.537	-0.039	0.374	-0.612	-0.318	-0.396	-0.420	-0.731	-0.155	0.296	0.336

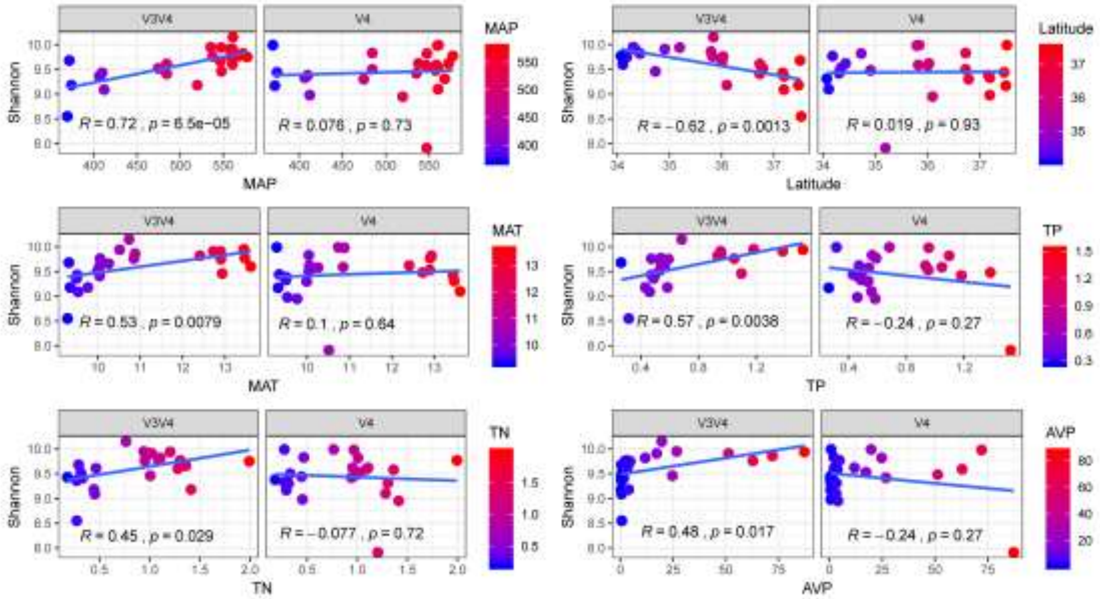

Fig. S1 The associations between bacterial diversity and environmental factors using linear regressions.

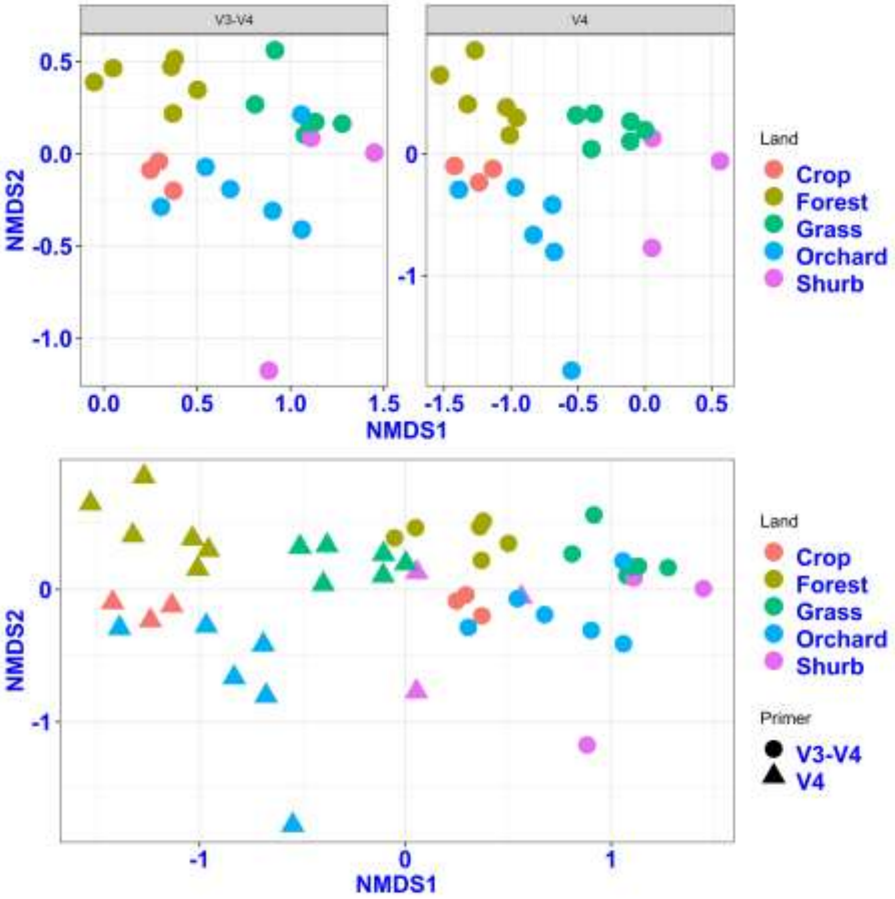

Fig. S2 The community structure under different land uses using NMDS plots

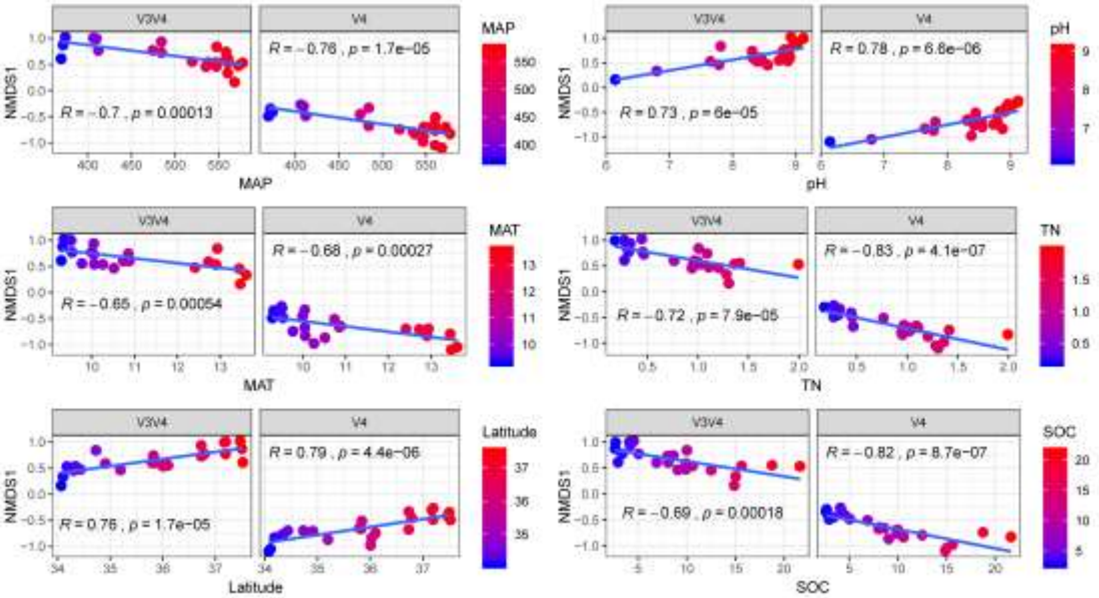

Fig. S3 The associations between bacterial community structure and environmental factors using linear regressions.

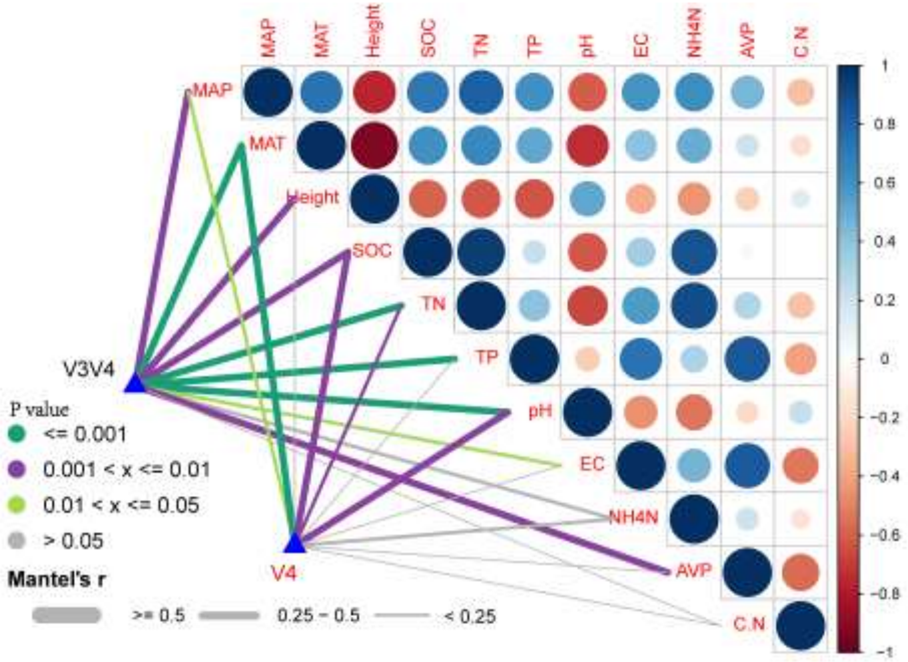

Fig. S4 The associations between community structure and environmental factors using mantel test. V3V4 means the bacterial community structure tested by the primer pairs of 338f/806r targeting V3-V4 region; and V4 means the bacterial community structure tested by the primer pairs of 520f/802r targeting V4 region.

Reference:

1. Liu D., Yang Y., An S., Wang H., Wang Y., The Biogeographical Distribution of Soil Bacterial Communities in the Loess Plateau as Revealed by High-Throughput Sequencing. *Frontiers in Microbiology*. 2018;9:2456.
